# Healthcare-associated Viral Gastroenteritis among Children in a Large Pediatric Hospital, United Kingdom

**DOI:** 10.3201/eid1601.090401

**Published:** 2010-01

**Authors:** Nigel A. Cunliffe, J. Angela Booth, Claire Elliot, Sharon J. Lowe, Will Sopwith, Nick Kitchin, Osamu Nakagomi, Toyoko Nakagomi, C. Anthony Hart, Martyn Regan

**Affiliations:** University of Liverpool, Liverpool, UK (N.A. Cunliffe, J.A. Booth, O. Nakagomi, T. Nakagomi, C.A. Hart); Royal Liverpool Children’s National Health Service Foundation Trust, Liverpool (N.A. Cunliffe, C. Elliot, S.J. Lowe, C.A. Hart); Health Protection Agency NW, Liverpool (C. Elliot, W. Sopwith, M. Regan); Sanofi Pasteur MSD, Maidenhead, UK (N. Kitchin); Nagasaki University, Nagasaki, Japan (O. Nakagomi, T. Nakagomi); 1 Deceased.

**Keywords:** Rotavirus, norovirus, nosocomial, gastroenteritis, molecular, vaccine, viruses, research, UK, children

## Abstract

Viruses are the major pathogens of community-acquired (CA) acute gastroenteritis (AGE) in children, but their role in healthcare-associated (HA) AGE is poorly understood. Children with AGE hospitalized at Alder Hey Children’s Hospital, Liverpool, UK, were enrolled over a 2-year period. AGE was classified as HA if diarrhea developed >48 hours after admission. Rotavirus, norovirus, adenovirus 40/41, astrovirus, and sapovirus were detected by PCR. A total of 225 children with HA-AGE and 351 with CA-AGE were enrolled in the study. HA viral gastroenteritis constituted one fifth of the diarrheal diseases among hospitalized children and commonly occurred in critical care areas. We detected >1 virus in 120 (53%) of HA-AGE cases; rotavirus (31%), norovirus (16%), and adenovirus 40/41 (15%) were the predominant viruses identified. Molecular evidence indicated rotaviruses and noroviruses were frequently introduced into the hospital from the community. Rotavirus vaccines could substantially reduce the incidence of HA-AGE in children.

## Abstract 2

Enteric viruses introduced from the community are major causes of these illnesses.

Enteric viruses are major etiologic agents of acute gastroenteritis (AGE) among infants and young children worldwide (*1*). Rotavirus is the most common single cause of severe diarrhea leading to dehydration and death (*2*). Other recognized viral causes of pediatric gastroenteritis include norovirus, astrovirus, enteric adenovirus (serotypes 40 and 41), and sapovirus (*1*).

Although studies of viral gastroenteritis in children have mainly focused on community-acquired (CA) infection, the importance of healthcare-associated (HA) rotavirus infection and the potential for its prevention by vaccination has been highlighted in several recent publications (*3**–**8*). Despite improved understanding of the disease impact and epidemiology of HA rotavirus gastroenteritis, many studies have been limited by inadequate design and methodology, short duration, and small size.

The value of molecular methods in defining the contribution of multiple viruses to pediatric diarrheal disease is being increasingly recognized (*9**,**10*). However, no studies have examined each of the known viral gastroenteritis agents among children with HA-AGE. In a 2-year prospective study in a large pediatric hospital, we examined the contribution to HA-AGE and CA-AGE of rotavirus and 4 additional enteric viruses using the most sensitive molecular detection methods available.

## Materials and Methods

### Study Setting

The study was conducted at the Royal Liverpool Children’s National Health Service Foundation Trust (Alder Hey Hospital). Alder Hey provides primary, secondary, and tertiary care facilities for >200,000 children each year and has ≈300 inpatient beds. General medicine, general surgery, and a range of specialist services including critical care, oncology, cardiac, and neurosurgery are provided.

### Enrollment Procedures

Children <16 years of age who were admitted with AGE during January 1, 2006–December 31, 2007, or those in whom AGE developed after hospitalization, were eligible for inclusion in the study. AGE was defined as diarrhea (≥3 loose, or looser-than-normal, stools in a 24-hour period), with or without vomiting, of <7 days’ duration. Gastroenteritis was considered HA if symptoms developed ≥48 hours after admission. Written informed consent was obtained from the child’s parent or guardian before enrollment.

### Collection of Clinical Data and Fecal Specimens

Study nurses identified case-patients by daily chart review. Clinical data were collected when patients were admitted and prospectively until hospital discharge. We used these data to calculate a severity score using a modified 20-point scoring system (Table 1). A stool sample was collected as soon as possible after onset of diarrhea. Fecal specimens were stored at −80°C until virus detection was undertaken. Rates of AGE were calculated by using total admission numbers obtained from hospital records.

**Table 1:** Modified 20-point scoring system used to evaluate severity of gastroenteritis (*11*)

Clinical sign or symptom	Points
Duration of diarrhea, d	
<2	1
2–4	2
>4	3
Maximum no. diarrheal stools in 24 h	
3	1
4–5	2
>5	3
Duration of vomiting, d	
No vomiting	0
1–2	2
>3	3
Maximum no. vomiting episodes in 24 h	
1	1
2	2
>3	3
Rehydration	
None	0
Oral	1
Intravenous	2
Fever, ºC	
<37.6	0
37.6ºC–38.5	2
>38.5	3
Behavior	
Normal	0
Lethargic/irritable	1
Convulsion	3

### Laboratory Analyses

#### Virus Detection

Nucleic acid was extracted from 10% fecal suspensions in phosphate buffered saline by using the RNeasy mini kit (QIAGEN, Crawley, UK). After reverse transcription–PCR (RT-PCR) was performed by using random hexamers, rotavirus, adenovirus 40/41, astrovirus, and sapovirus were each detected by conventional PCR using virus-specific primers, agarose gel electrophoresis, and ethidium bromide staining (*9**,**12*). Norovirus was detected by using a modification of the real-time PCR method of Kageyama et al. (*13*) as described by Amar et al. (*9*).

#### Molecular Characterization of Rotaviruses and Noroviruses

Rotavirus viral protein (VP) 7 (G) and VP4 (P) were genotyped by using multiplex, heminested RT-PCR (*14**,**15*). The dsRNA genomes of genotype P[8],G1 rotavirus strains were examined by polyacrylamide gel electrophoresis (PAGE). The VP7 gene of P[8],G1 rotavirus strains was amplified by RT-PCR using primers 9con1 and 9con2 (*15*). The capsid region of Norovirus genogroup II strains was amplified by RT-PCR using primers G2SKF and G2SKR (*16*). All RT-PCR amplification products were purified by using Micro-spin columns (GE Healthcare, Buckinghamshire, UK), and sequenced by Cogenics (Hope End, Essex, UK). Phylogenetic trees were constructed according to the neighbor-joining method (*17*) in the ClustalW software package (*18*).

## Results

### Subject Enrollment and Case Classification

AGE in 669 children met the case definition for investigation during the study. We excluded 93 children from analysis because of failure to obtain fecal specimens (80 children), consent refusal/withdrawal (12), and age >16 years (1). Of the remaining 576 children with AGE, 351 cases (61%) were determined to be CA and 225 (39%) were HA.

### Virus Detection Rates

During the study period, ≥1 viruses were detected in 339 (59%) of 576 specimens. Rotavirus was identified most frequently (38% of all AGE cases), followed by norovirus (16%), adenovirus 40/41 (14%), astrovirus (5%), and sapovirus (5%) (Table 2). The order of detection of each virus did not differ between CA- and HA-AGE cases. We detected >1 virus in 120 (53%) of 225 HA-AGE cases. Rotavirus was detected in 70 (58%) of 120 children with HA-AGE in whom a virus was identified.

**Table 2:** Comparison of types of viruses detected among children hospitalized at Alder Hey Hospital for HA-AGE versus CA-AGE, Liverpool, UK, 2006–2007*

Virus	No. (%) case-patients	
	HA-AGE, n = 225	CA-AGE, n = 351
Rotavirus	70 (31)	150 (43)
Norovirus	36 (16)	54 (15)
Adenovirus 40/41	34 (15)	49 (14)
Astrovirus	12 (5)	16 (5)
Sapovirus	5 (2)	22 (6)
Any virus detected	120 (53)	219 (62)
No virus detected	105 (47)	132 (38)

*CA-AGE, community-acquired acute gastroenteritis; HA-AGE, healthcare-associated acute gastroenteritis.

In 98 (17%) of 576 children with AGE, >1 virus was detected; this proportion did not significantly differ between CA (65/351 cases, 19%) and HA (33/225, 15%) infection. Rotavirus was the agent least likely to be identified as a mixed infection; it occurred as the only virus in 144 (65%) of 220 AGE cases in which it was identified. This proportion did not differ between patients with CA (100/150, 66%) and HA (44/70, 62%) rotavirus infection (Table 3). Sapovirus was least likely to be identified as a sole infection, with only 4 (15%) of 27 cases involving no other virus.

**Table 3:** Mixed virus infections among children hospitalized at Alder Hey Hospital, by types of viruses detected, Liverpool, UK, 2006–2007*

Virus	Total no. case-patients	No. case-patients also infected with					
		Rotavirus	Norovirus	Adenovirus 40/41	Astrovirus	Sapovirus	>2 viruses
Rotavirus	76		14	16	3	10	7
Norovirus	44	6		6	2	3	4
Adenovirus 40/41	48	10	2		1	2	5
Astrovirus	16	4	2	2		0	2
Sapovirus	23	1	1	0	0		4
>2 viruses	37	5	4	4	0	2	

*Enumerates case-patients in whom >1 virus was identified. Numbers above diagonal represent community-acquired acute gastroenteritis (AGE); numbers below diagonal represent healthcare-associated AGE. NHS, National Health Service.

### Age Distribution of Case-patients

The median age of children with all-cause CA-AGE (10 mo, range 1–180 mo) did not differ from children with all-cause HA-AGE (12 mo, range 1–192 mo). Among case-patients, the median age of children with rotavirus infection (11 mo, range 1–192 mo) was similar to children excreting norovirus (10 mo, range 1–180 mo); adenovirus 40/41 (10 mo, 1–180 mo); astrovirus (9 mo, 1–180 mo); and sapovirus (12 mo, 1–96 mo). The median age of children excreting any virus did not differ according to exposure setting (data not shown).

### Severity of Viral Gastroenteritis

The severity of AGE caused by infection only with rotavirus or norovirus was greater than with other viruses for CA and HA infections (Table 4). The proportion of children with severe gastroenteritis (defined as severity score ≥11) was greatest with rotavirus (42/144, 29%) and norovirus (12/46, 26%) and lowest for children in whom virus was not detected (39/237, 16%). Rotavirus was associated with severe illness in 36% of HA-AGE cases in which it was identified as the only infection, not significantly different to the proportion (24%) of HA-AGE judged to be severe as a consequence of single norovirus infection (p = 0.400 by Fisher exact test). Overall, 28% of HA-viral gastroenteritis patients were defined as having severe disease.

**Table 4:** Median AGE severity scores among children hospitalized at Alder Hey Hospital for HA-AGE versus CA-AGE, by types of viruses detected, Liverpool, UK, 2006–2007*

Virus	HA-AGE, n = 225 (range)	CA-AGE, n = 351 (range)
Rotavirus	8 (3–17)	8 (2–16)
Norovirus	8 (3–14)	9 (2–15)
Adenovirus 40/41	6 (3–12)	6 (4–14)
Astrovirus	7.5 (6–9)	7.5 (5–11)
Sapovirus	11 (11)	5 (4–8)
Any virus detected	8 (3–17)	8 (2–16)
No virus detected	7 (3–15)	7 (2–15)

*CA-AGE, community-acquired acute gastroenteritis; HA-AGE, healthcare-associated acute gastroenteritis.

### Molecular Epidemiology of Virus Infections

Rotavirus genotype could be determined for 93 (62%) of 150 CA-AGE cases and for 23 (33%) of 70 HA-AGE cases (rotaviruses that could not be genotyped were considered to contain insufficient virus load in stool [data not shown]). Distribution of rotavirus strains did not differ between CA and HA cases, with the P[8],G1 strain predominating in each. Thus, of rotaviruses genotyped from 93 patients with CA rotavirus, genotypes of 62 gastroenteritis patients were P[8], G1; 7 were P[8], G3; 4 were P[4], G2; 18 were P[8], G9; and 1 was P[8], G4; 1 strain could not be G or P typed. Of rotaviruses genotyped from 23 patients with HA rotavirus gastroenteritis, 19 were P[8], G1; 1 was P[8], G3; 2 were P[4], G2; and 1 was P[4], G1 + G2.

**Figure 1:**
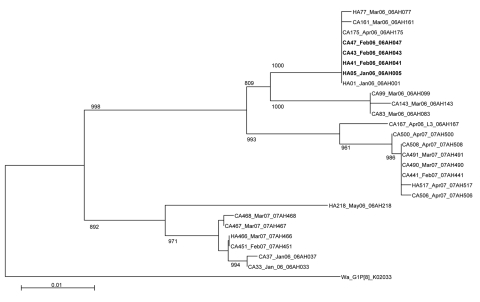
Phylogenetic tree based on viral protein (VP) 7 nucleotide sequences of serotype G1 rotavirus strains from Royal Liverpool Children’s National Health Service Foundation Trust (Alder Hey Hospital), Liverpool, UK. For each strain the source (healthcare-associated [HA] or community-acquired [CA]), specimen number, month/year of detection, and name of the strain is indicated. Reference G1P[8] strain Wa is included. Horizontal lengths are proportional to the genetic distance calculated with Kimura’s 2-parameter method. Scale bar shows genetic distance expressed as nucleotide substitutions per site. Bootstrap probabilities >80% (>800 of 1,000 pseudoreplicate trials) are indicated at each node. Hatched VP7 sequences are from strains whose electropherotypes shared an identical L1 pattern. The VP7 nucleotide sequences used in the tree have been deposited under the strain name and accession number (in parentheses) as follows; 06AH001 (FJ797814), 06AH005 (FJ797815), 06AH033 (FJ797816), 06AH037 (FJ797817), 06AH041 (FJ797818), 06AH043 (FJ797819), 06AH047 (FJ797820), 06AH077 (FJ797821), 06AH083 (FJ797822), 06AH099 (FJ797823), 06AH143 (FJ797824), 06AH161 (FJ797825), 06AH167 (FJ797826), 06AH175 (FJ797827), 06AH218 (FJ797828), 07AH441 (FJ797829), 07AH451 (797830), 07AH466 (FJ797831), 07AH467 (FJ797832), 07AH468 (FJ797833) , 07AH490 (FJ797834), 07AH491 (FJ797835), 07AH500 (FJ797836), 07AH506 (FJ797837), 07AH508 (FJ797838), 07AH517 (FJ797839).

**Figure 2:**
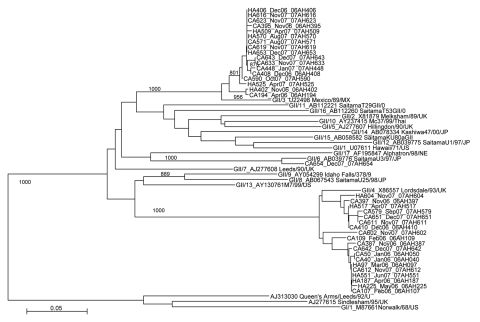
Phylogenetic tree based on 387 nucleotide sequences of the 5′ end of open reading frame 2 (encoding viral protein 1) of norovirus strains from Royal Liverpool Children’s National Health Service Foundation Trust (Alder Hey Hospital), Liverpool, UK. For each strain the source (healthcare-associated [HA] or community-acquired [CA]), specimen number, month/year of detection, and the name of the strain is indicated. Reference strains included on the tree are GII/1 U07611 Hawaii/71/US, GII/2 X81879 Melksham/89/UK, GII/3 U22498 Mexico/89/MX, GII/4 X86557 Lordsdale/93/UK, GII/5 AJ277607 Hillingdon/90/UK, GII/6AB039776 SaitamaU3/97/JP, GII/7 AJ277608 Leeds/90/UK, GII/8 AB067543 SaitamaU25/98/JP, GII/9 AY054299 IdahoFalls/378/96/US, GII/10 AY237415 Mc37/99/Thai, GII/11 AB112221 SaitamaT29GII/01/JP, GII/12 AB039775 SaitamaU1/97/JP, GII/13 AY130761 M7/99/US, GII/14 AB078334 Kashiwa47/00/JP, GII/15 AB058582 SaitamaKu80aGII/99/JP, GII/16 AB112260 SaitamaT53GII/02/JP, GII/17 AF195847 Alphatron/98/NE, GI/1 M87661 Norwalk/68/US, AJ313030 Queen’sArms/Leeds/92/UK, AJ277615 Sindlesham/95/UK. Horizontal lengths are proportional to the genetic distance calculated with the Kimura 2-parameter method. Scale bar shows genetic distance expressed as nucleotide substitutions per site. Bootstrap probabilities >80% (>800 of 1,000 pseudoreplicate trials) are indicated at each node. The nucleotide sequences used in the tree have been deposited under the strain name and accession number (in parentheses) as follows; 06AH107 (FJ797840), 06AH109 (FJ797841), 06AH187 (FJ797842), 06AH194 (FJ797843), 06AH225 (FJ797844), 06AH387 (FJ797845), 06AH395 (FJ797846), 06AH397 (FJ797847), 06AH040 (FJ797848), 06AH402 (FJ797849), 06AH406 (FJ797850), 06AH408 (FJ797851), 06AH410 (FJ797852), 07AH448 (FJ797853), 06AH050 (FJ797854), 07AH509 (FJ797855), 07AH517 (FJ797856) 07AH525 (FJ797857), 07AH551 (FJ797858), 07AH570 (FJ797859), 07AH571 (FJ797860), 07AH579 (FJ797861), 07AH590 (FJ797862), 07AH602 (FJ797863), 07AH604 (FJ797864), 07AH611 (FJ797865), 07AH612 (FJ797866), 07AH616 (FJ797867), 07AH619 (FJ797868), 07AH623 (FJ797869), 07AH633 (FJ797870), 07AH642 (FJ797871), 07AH643 (FJ797872), 07AH651 (FJ797873), 07AH653 (FJ797874), 07AH654 (FJ797875), 06AH097 (FJ797876).

To explore whether HA-AGE cases were associated with similar or identical rotavirus strains causing CA-AGE, we examined P[8], G1 rotaviruses further by PAGE and by VP7 gene sequencing. Three distinct electropherotypes (L1, L2, and L3) were identified among 19 HA P[8], G1 strains, and each of these electropherotypes also was recognized among 7 electropherotypes assigned to 62 CA P[8], G1 strains (data not shown). Similarly, all except 2 VP7 nucleotide sequences from HA-AGE rotavirus infections clustered with corresponding sequences from CA strains with which they shared almost identical or identical nucleotide sequence (Figure 1). For noroviruses, all except 1 HA-AGE norovirus sequences were identical, or almost identical, to those derived from CA strains (Figure 2).

### Hospital Distribution of Viral Gastroenteritis Cases

**Figure 3:**
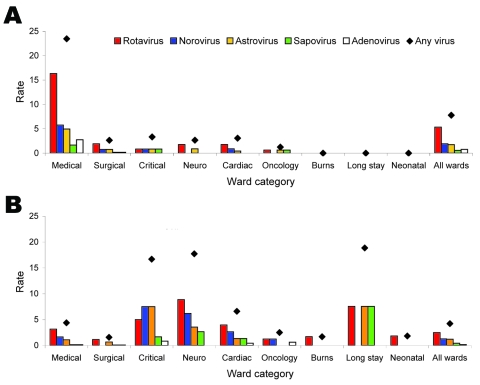
Distribution of case-patients with A) community-acquired versus B) healthcare-associated acute gastroenteritis (AGE) in whom a virus was detected, by ward category and virus detected, Alder Hey Hospital, Liverpool, UK, 2006–2007. Rates were calculated as numbers of cases per 1,000 admissions to each ward throughout the study and are shown for each virus tested, with a comparison of all cases where at least 1 virus was detected (diamonds).

Although 274 (94%) of 291 patients with CA viral gastroenteritis were located in acute general medical and surgical wards, 88 (56%) of 157 with HA viral gastroenteritis were located elsewhere in the hospital. In particular, the highest rates of HA viral gastroenteritis infection were noted in critical care units (intensive care unit and high dependency unit), neurology, cardiology, and long stay wards where children with chronic conditions who have complex healthcare needs are patients (although this represents <5 case-patients) (Figure 3). Median duration of hospital stay before onset of symptoms among children with HA viral gastroenteritis was 8 days (range 2–1,365 days); two thirds of infections occurred at least 1 week after hospital admission.

## Discussion

In our study, one fifth of AGE among children within a large pediatric hospital was of viral origin and acquired within the healthcare setting. Furthermore, at least 1 virus was detected in >50% of HA-AGE patients, and in 28%, >1 virus was identified. HA viral gastroenteritis is a major infection control issue in hospital pediatric wards (*19*). Our study systematically detected each of the 5 established causes of viral gastroenteritis.

Rotavirus was the most common virus identified among children with CA- and HA-AGE (43% and 31%, respectively). It was also the virus most likely to occur alone and was the virus with which severe symptoms were most often associated. A recent review of European studies identified rotavirus in 31%–87% of all-cause HA-AGE, reflecting the differences in methods used in individual studies (*4*). HA rotavirus infections in our study accounted for 32% of all rotavirus infections identified. This finding is consistent with 2 recent retrospective studies showing that 21% (*20*) and 36% (*21*) of rotavirus gastroenteritis cases were HA and 2 reviews documenting that 14%–51% of rotavirus infections among hospitalized children were HA (*3**,**5*). Therefore, the impact of HA rotavirus gastroenteritis in major pediatric hospitals has not decreased since the first major study of this subject published nearly 20 years ago (*22*).

With the availability and application of molecular assays capable of detecting a broad range of norovirus genotypes, norovirus is now recognized as a major cause of sporadic CA-AGE in children (*23**–**25*). We document that norovirus was the second most common virus identified (after rotavirus) among children with HA-AGE (detected in 16% of HA-AGE patients and in 24% of HA-AGE patients in which a single virus was identified). Furthermore, norovirus was associated with severe illness in a substantial percentage (24%) of HA-AGE patients in whom it was identified as a sole infection, a percentage similar to that obtained for rotavirus (36%).

Previous studies have investigated adenovirus 40/41 in CA-AGE, and we systematically looked for it in children with HA-AGE. Adenovirus 40/41 was the third most commonly identified virus in our study among children with CA- and HA-AGE (detected in 14% and 15% of patients, respectively). Studies that have investigated for adenovirus 40/41 among children with CA-AGE by using antigen-based detection methods have generally reported detection rates of ≈5% (*1*). We used molecular methods, therefore suggesting a greater role for adenovirus 40/41 in CA-AGE than has been previously appreciated, and now indicating it also plays a prominent role in HA-AGE. Notably, adenovirus 40/41 was also the third most commonly identified enteric virus in a recent study in East Anglia, UK where it was detected by PCR in 9.6% of all children examined (*10*). Astroviruses and sapoviruses were much less frequently identified in our study, despite the application of molecular methods.

With the use of molecular methods to detect 5 established viral agents of gastroenteritis, we demonstrated that 15% of HA-AGE patients contained >1 virus, emphasizing the value of the simultaneous examination for multiple viruses. Thus, in a recent community-based study in the United Kingdom that examined for each of these 5 viruses using molecular assays, mixed virus infections were identified in 11.7% of case-patients (*24*). In almost 50% of the norovirus infections in our study, an additional virus was identified (most commonly rotavirus). Although we found no evidence of increased disease severity among rotavirus–norovirus co-infections (data not shown), our data clearly demonstrate the frequency with which both viruses cocirculate with the potential for nosocomial spread. Dual norovirus–rotavirus co-infections were also commonly recognized in a recent study of hospitalized children in Italy, in whom the severity of illness was higher than with norovirus infection alone (*26*). Similar to the observations made for norovirus, additional virus infections were commonly identified among case-patients excreting adenovirus 40/41, in whom more than half had additional viruses.

Although each of the enteric viruses detected in this study is firmly established as a gastroenteritis pathogen, the detection of a virus in a child with AGE does not necessarily imply causation. This is most clearly relevant in the context of co-infections including mixed virus infections, where >1 detected organisms may not be primarily responsible for diarrhea. Although we did not exhaustively and systematically search for other potential infective causes of diarrhea, we identified enteropathogenic bacteria and parasites in <2% of specimens examined, which suggests that they were not major causes of diarrhea in our study population (data not shown). Additionally, although detection of a virus by a sensitive molecular method may be more likely than a less sensitive antigen-based assay to indicate subclinical infection (*27*), prolonged rotavirus shedding demonstrated by RT-PCR was associated with symptoms of diarrhea in a longitudinal study of children recovering from severe rotavirus infection (*28*). Because molecular-based assays are widely accepted as the preferred detection method for norovirus, but not for other gastroenteritis viruses, application in this study of molecular methods that likely have similar (high) levels of sensitivity for detection of all 5 gastroenteritis viruses allowed more appropriate recognition of the extent of virus circulation within the hospital. Finally, although a stool sample obtained from a patient at hospital admission would be required to conclusively categorize a viral gastroenteritis episode as CA or HA, two thirds of these infections occurred >1 weeks after admission, and a 48-hour symptom-based definition has been used extensively in previous studies (*3**–**5*).

The entry and spread of enteric viruses into and within pediatric healthcare settings is not completely understood. Several studies have indicated that multiple rotavirus introductions from the community into the hospital and subsequent spread between patients accounts for most HA rotavirus infections because of the diversity among strains recovered from hospitalized children and similarity at the genotype level between viruses circulating within the community and hospital (*20**,**29**,**30*). Similarly, a recent study that described frequent, brief clusters of norovirus gastroenteritis among pediatric inpatients attributed these findings to frequent introduction of noroviruses into the hospital from the community (*31*). For both rotaviruses and noroviruses, however, previous studies have not provided direct evidence that strains detected among hospitalized children originated in the community. In this respect, our study provides strong molecular epidemiologic support, at the nucleotide level, for the hypothesis that rotaviruses and noroviruses in the community are the sources of HA infection. Thus, rotaviruses (judged by VP7 sequence), and noroviruses (examined by sequence of the capsid region) detected among HA-AGE cases were similar or identical to corresponding viruses detected in children with CA-AGE.

In contrast to CA-AGE cases, the highest rates of HA- viral enteric infections did not occur on the acute medical and surgical wards but instead in the critical care areas, neurologic, cardiac, and long stay wards where chronically ill children are patients. We observed this trend for each of the 3 major viruses identified (rotavirus, norovirus, and adenovirus 40/41). Although we noticed differences in the hospital distribution of HA-AGE cases, the ages of children with HA-AGE did not differ significantly from children with CA-AGE. We did, however, find evidence for severe disease among 28% of children with HA viral gastroenteritis in our study; 22% of such children had an underlying medical condition (data not shown). Given that CA viruses are likely to be the source of HA viral gastroenteritis, standard hospital infection prevention measures (e.g., isolation of children with AGE) should be emphasized (*32*). Hand washing by staff before patient contact may be particularly important because asymptomatic staff members could be a source of HA rotavirus infection (*33*) and hand washing has been shown to reduce the rate for nosocomial rotavirus infection (*34*). However, because HA rotavirus infection remains a substantial clinical problem ≈20 years after publication of a seminal study on the subject (*22*), even stringent infection control precautions are likely to be insufficient to greatly reduce the problem. In the context of rotavirus, the major pathogen responsible for CA- and HA-AGE, rotavirus vaccines offer a further opportunity to reduce the impact of HA-AGE. Routine rotavirus vaccination of children in the community is expected to greatly reduce the number of CA, rotavirus-associated hospitalizations (*35**,**36*), thereby reducing the hospital rotavirus reservoir and HA rotavirus gastroenteritis. The prevention of HA rotavirus gastroenteritis should represent an important secondary goal of rotavirus vaccines (*3**,**6*).

In conclusion, we have demonstrated that viruses accounted for more than half the cases of HA-AGE in a large pediatric hospital and resulted from the frequent introduction of gastroenteritis viruses from the community. Because rotavirus is the single most common pathogen, introduction of a rotavirus vaccine into childhood immunization programs is expected to substantially reduce the incidence of CA and HA rotavirus gastroenteritis in hospitals.
